# Cytokines and chemokines skin gene expression in correlation with immune cells in blood and severity in equine insect bite hypersensitivity

**DOI:** 10.3389/fimmu.2024.1414891

**Published:** 2024-07-15

**Authors:** Fadi Jebbawi, Alex Chemnitzer, Macsmeila Dietrich, Stanislav Pantelyushin, Juwela Lam, Tanya Rhiner, Giulia Keller, Nina Waldern, Fabia Canonica, Antonia Fettelschoss-Gabriel

**Affiliations:** ^1^ Department of Dermatology, University Hospital Zurich, Schlieren, Switzerland; ^2^ Faculty of Medicine, University of Zurich, Zurich, Switzerland; ^3^ Evax AG, Guntershausen, Switzerland; ^4^ Equine Department, Vetsuisse Faculty, University of Zurich, Zurich, Switzerland

**Keywords:** biomarker, insect bite hypersensitivity, skin, allergy, equine, cytokines

## Abstract

**Background:**

Insect bite hypersensitivity (IBH) is the most frequent skin allergy of horses and is highly debilitating, especially in the chronic phase. IBH is caused by IgE-mediated hypersensitivity reactions to culicoides midge bites and an imbalanced immune response that reduces the welfare of affected horses.

**Objective:**

In the present study, we investigated the pathological mechanisms of IBH, aiming to understand the immune cell modulation in acute allergic skin lesions of IBH horses with the goal of finding possible biomarkers for a diagnostic approach to monitor treatment success.

**Methods:**

By qPCR, we quantified the gene expression of cytokines, chemokines, and immune receptors in skin punch biopsies of IBH with different severity levels and healthy horses simultaneously in tandem with the analysis of immune cell counts in the blood.

**Results:**

Our data show an increase in blood eosinophils, monocytes, and basophils with a concomitant, significant increase in associated cytokine, chemokine, and immune cell receptor mRNA expression levels in the lesional skin of IBH horses. Moreover, IL-5Ra, CCR5, IFN-γ, and IL-31Ra were strongly associated with IBH severity, while IL-31 and IL-33 were rather associated with a milder form of IBH. In addition, our data show a strong correlation of basophil cell count in blood with IL-31Ra, IL-5, IL-5Ra, IFN-γ, HRH2, HRH4, CCR3, CCR5, IL-12b, IL-10, IL-1β, and CCL26 mRNA expression in skin punch biopsies of IBH horses.

**Conclusion:**

In summary, several cytokines and chemokines have been found to be associated with disease severity, hence contributing to IBH pathology. These molecules can be used as potential biomarkers to monitor the onset and progression of the disease or even to evaluate and monitor the efficacy of new therapeutic treatments for IBH skin allergy. To our knowledge, this is the first study that investigated immune cells together with a large set of genes related to their biological function, including correlation to disease severity, in a large cohort of healthy and IBH horses.

## 1 Introduction

Similar to humans, animals also suffer from different forms of allergy when exposed to allergens. Common hallmarks of skin allergies are an imbalanced immune reaction causing severe symptoms that reduce the welfare of affected animals. Insect bite hypersensitivity (IBH) is a very frequent allergic disease in horses caused by insect bites, more specifically by the salivary gland proteins of biting midges, mainly the culicoides species (1–5). IBH was earlier classified as a type I allergy (6, 7), whereas characteristics of delayed type IV hypersensitivity have been suggested (5, 8–10). Hence, it is not surprising that IBH horses show an increase of culicoides allergen-specific IgE levels in serum when compared to healthy horses (11–13). Moreover, allergen-specific IgE levels are raised concurrently with the initial onset of clinical signs of IBH, as observed in a study in horses imported from Iceland to a culicoides-infested area of Europe (14). However, it was shown that allergen-specific serum IgE cannot be used as a predictor of IBH (14) and that also there was no correlation of disease severity with *Culicoides*-specific IgE when using *Culicoides nubeculosus* whole-body extract (15). Affected horses develop itchy skin lesions along the dorsal and ventral bodyline with an “eczema-like” morphology, with hyperkeratotic scales, that become worse with scratching, resulting in exudate, crusts, swelling, and lichenification. Moreover, IBH horses are susceptible to developing allergic reactions elsewhere and have a higher risk for airway hyperreactivity (16), which could potentially develop into equine asthma in the future (13). That turn of events can become life-threatening for horses and is a burden for horse owners.

The major players in IBH allergy are numerous, such as T helper type 2 (Th2), regulatory T cells (Tregs), eosinophils, epithelial cells, keratinocytes, mast cells, monocytes, and basophils, which all have been postulated to be involved in the pathological immune responses in IBH (9). The aim of this study is to shed light on the pathological mechanisms of IBH during the early and chronic phases of the disease. There is a necessity to develop a diagnostic approach and ideally identify biomarkers to follow the onset of disease, monitor progression and severity, and potentially monitor the efficacy of a treatment for IBH in the future. Thus, we investigated a gene expression panel of cytokines, chemokines, and receptors in correlation with the major players in IBH on a large number of horses in tandem with immune cell counts in blood, taking healthy horses as control and IBH horses by severity. The skin sample requires only a small 2-mm skin punch biopsy and therefore is a big advantage when compared to more invasive 6-mm biopsies for histology. To our knowledge, this is the first study that addressed the modulation of various immune cell-related genes in the skin by individual qPCR in correlation to immune cells in blood and disease severity. Moreover, the study suggested top molecules significantly differing in healthy versus mild versus moderate/severe IBH horses and, hence, possibly serving as biomarkers.

## 2 Materials and methods

### 2.1 Ethics

All study horses were privately held by their owners. All horse owners provided signed informed consent. All interventions were approved by the cantonal veterinary authorities (License 33558). The horses were screened in the IBH season (**Supplementary Figure S1**) prior to the study. All “IBH horses” showed clinical signs of IBH lesions during the entire season at dorsal and ventral body lines with typical characteristics such as hair loss, scales, crust, excoriations, lichenification, and swelling. All horses were dewormed regularly. Horse groups included the following: IBH with lesional biopsies and healthy controls. Skin punch biopsies were collected from the mane, head, breast, flank, and knee of IBH horses and only from the mane of healthy horses. Moreover, IBH horses were divided into mild (M) and moderate/severe (M/S) groups with lesion scores ≤100 and >100, respectively.

### 2.2 Punch biopsies

Punch biopsies (2 mm) from lesional (n = 34) skin of IBH-affected, mild (M) (n = 6), and moderate/severe (M/S) (n = 28) IBH horses (**Supplementary Table 1**), as well from healthy (H) (n = 24) skin of non-IBH horses were collected into RNAlater™ stabilization solution (Thermo Fisher, Waltham, MA, USA) for RNA extraction as described previously (17).

### 2.3 Blood analysis by IDEXX Diavet

Blood analysis was conducted on a large cohort of horses from April to October, during the warm season. Immune cell count and differential blood analysis on samples from healthy (n = 33) and IBH (n = 80) [M (n = 31) and M/S (n = 49)] horses were conducted using fresh ethylenediaminetetraacetic acid (EDTA) blood, as measured by IDEXX Diavet.

### 2.4 Blood collection for PBMC isolation and *in vitro* restimulation

A subgroup of horses was randomly selected for an *in vitro* stimulation experiment comprising healthy (n = 22) and IBH {n = 40 [M (n = 29) and M/S (n = 11)]} horses. Blood was collected for peripheral blood mononuclear cell (PBMC) isolation using NH Sodium Heparin VACUETTE^®^ containers (Greiner Bio-One, Kremsmuenster, Austria).

### 2.5 IBH lesion scoring

The methodology is described in Reference (15).

### 2.6 PBMC stimulation with culicoides *in vitro*

Density gradient centrifugation (Ficoll-Paque™ Plus, Cytiva, Marlborough, MA, USA; cat. GE17–1440-03) was used to isolate PBMCs from diluted heparinized blood in phosphate-buffered saline (PBS) 1× (Thermo Fisher, cat. 10010023) in SepMate 50-mL tubes (STEMCELL, Vancouver, BC, Canada; cat. 85460), according to the manufacturer’s instructions. PBMCs were counted; resuspended in RPMI GlutaMAX 1640 medium (Thermo Fisher, cat. 72400021) complete with 10% fetal bovine serum (FBS), 1% PenStrep (Thermo Fisher, cat. 15140122), 1× NEAA (Thermo Fisher, cat. 11140050), and 1× sodium pyruvate (Thermo Fisher, cat. 11360070); and stimulated for 24 h with whole-body extract (WBE) of *C. nubeculosus* (10 μg/mL, Stallergenes Greer, Baar, Switzerland; cat. XPB69X1A2.5) or medium alone. Cells were harvested by centrifugation and processed by qPCR using the Cells-to-Ct kit (Thermo Fisher, cat. A35381) according to the manufacturer’s instructions.

### 2.7 Total RNA extraction and qPCR

Total RNA was extracted from skin punch biopsies using TRIzol total RNA extraction (Sigma, St. Louis, MO, USA; cat. T9424). The concentration was quantified using a NanoDrop Spectrophotometer. To eliminate residual DNA, 1,000 ng of RNA was digested using DNAse I (Ambion, Austin, TX, USA; cat. AM2222). The purified RNA was then transcribed into cDNA using PrimeScript™ RT kit (Takara Bio, Mountain View, CA, USA; cat. RR037A) according to the manufacturer’s instructions.

Real-time PCR quantitative mRNA analyses were performed on the Viia 7 instrument using Fast SYBR green master mix (Thermo Fisher, 4385612). Data were normalized by subtracting the Ct of the housekeeping gene GAPDH from the Ct of the gene of interest, and the difference (ΔCT) was analyzed as 2^−ΔCT^. The primers used for RT-qPCR are listed in **Supplementary Table 2**.

### 2.8 Statistics

Data are presented as mean ± standard error of the mean (SEM). The significance of the differences between several groups was determined by the Kruskal–Wallis test followed by Dunn’s Multiple Comparison post-test. Significant differences between compared pair groups were calculated using the Mann–Whitney test. No outliers were excluded. The correlation of simultaneous blood cell differential counts and biopsy qPCR data of healthy (n = 24) and IBH (n = 31) horses was assessed by Spearman’s correlation two-tailed statistic test. The receiver operating characteristic (ROC) curve is a commonly used tool for evaluating performance by calculating the area under the curve (AUC). ROC analysis is independent of label distribution, which makes it a valuable tool commonly used in various applications including disease prediction (18, 19). ROC analysis was employed to assess the diagnostic efficiency of the diagnostic gene mRNA investigated in this study using GraphPad Prism. *p < 0.05, **p < 0.01, ***p < 0.001, and ****p < 0.0001 indicate significant differences. GraphPad Prism software (version 9, GraphPad, La Jolla, CA, USA) was used for statistical analysis.

## 3 Results

### 3.1 Immune cell differential counts in blood of healthy and IBH horses

Our results showed a significant increase in monocyte (1.15×), eosinophil (1.99×), and basophil (1.13×) cell counts in the blood of IBH horses when compared to healthy horses, while no significant difference in neutrophils and lymphocytes was observed (**Supplementary Figure S2A**).

**Figure 1:**
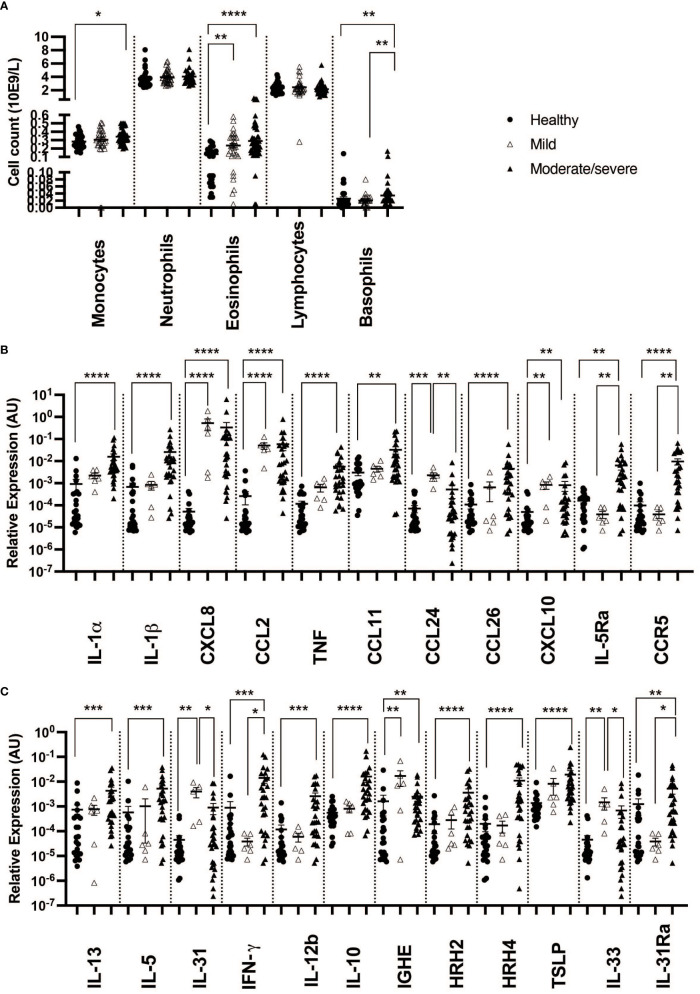
Differential blood counts and gene expression in skin punch biopsies from healthy and insect bite hypersensitivity (IBH) horses in relation to disease severity. Differential blood cells and skin punch biopsies from healthy skin (H) of healthy horses (n = 24 biopsies, n = 33 blood) and IBH horses (n = 34 lesional biopsies, n = 80 blood) divided into mild (M) (n = 6 biopsies, n = 31 blood) and moderate/severe (n = 28 biopsies, n = 49 blood) were collected simultaneously during IBH season with high symptoms in August. **(A)** Counts of monocytes, neutrophils, eosinophils, lymphocytes, and basophils in blood of healthy and IBH horses. Relative mRNA expression of inflammatory genes. **(B)** Relative mRNA expression of IL-1α, IL-1β, CXCL8, CCL2, TNF, CCL11, CCL24, CCL26, CXCL10, IL-5Ra, CCR3, and CCR5. **(C)** Relative mRNA expression of Th1, Th2, Treg-related genes IL-4, IL-13, IL-5, IL-31, IFN-γ, IL-12b, IL-10, IGHE, histamine receptor (HR) H2, HRH4, TSLP, IL-33, and IL-31Ra. *P < 0.05, **P < 0.01, ***P < 0.001, ****P < 0.0001.

When taking IBH severity into account, monocyte cell counts were found to increase when comparing moderate/severe cases to healthy controls. In addition, a significant increase in eosinophil counts was observed in the blood of both severity groups when compared to healthy horses. Also, basophil cell counts of moderate/severe IBH horses were significantly increased when compared to healthy and mild IBH horses. Lymphocyte and neutrophil cell counts remained comparable among IBH severity groups and healthy horses (**Figure 1A**).

### 3.2 Cytokines, chemokines, and receptors in healthy and IBH skin biopsies

#### 3.2.1 Pro-inflammatory cytokines and chemokines in skin of IBH horses

**Figure 2:**
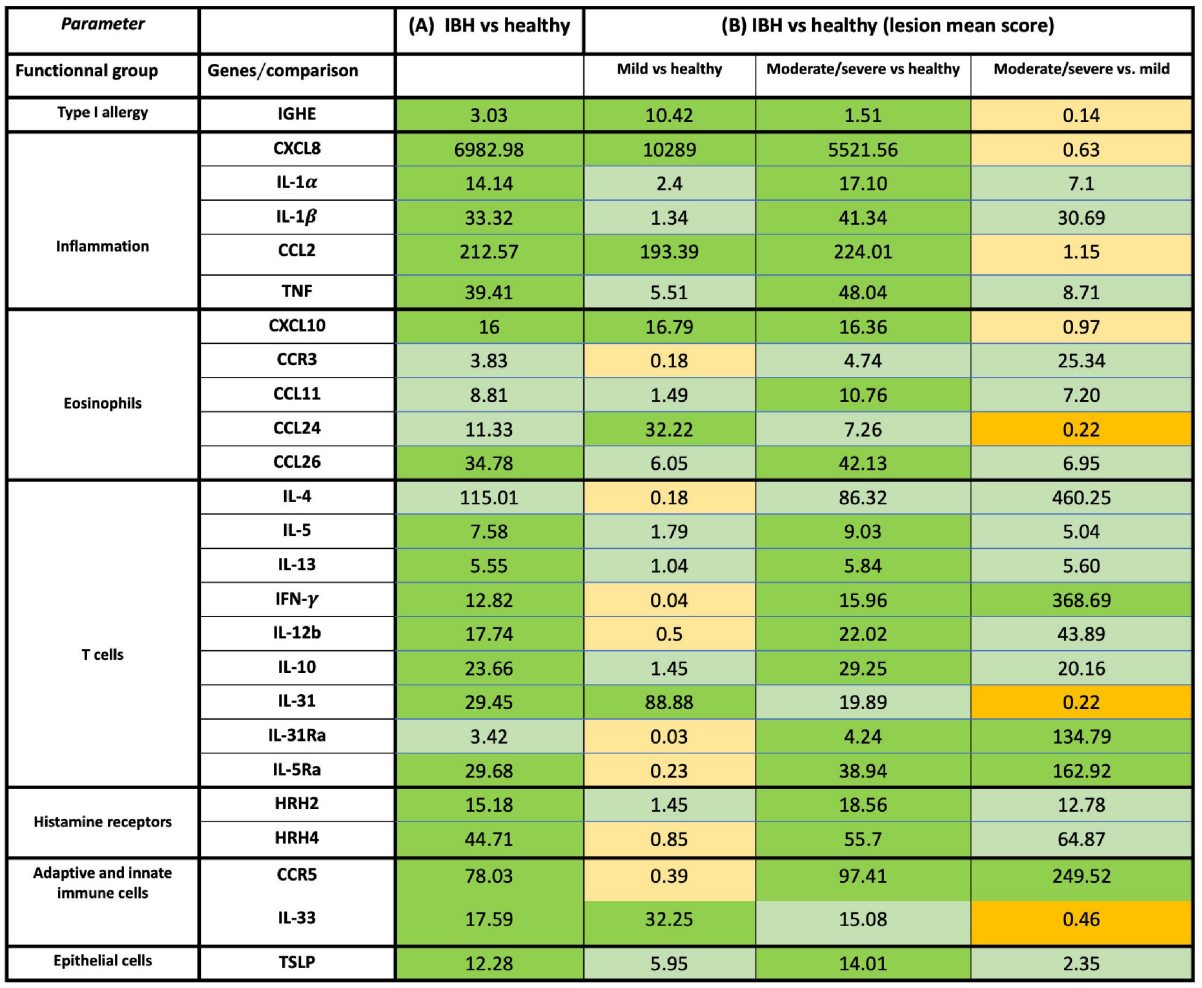
Overview of mRNA fold-change gene expression of cytokines, chemokines, and receptors in skin of healthy and insect bite hypersensitivity (IBH) horses. The genes were classified into specific functional groups based on their biological role in allergy. **(A)** Fold-change expression in IBH lesional (L) (n = 34) vs. healthy (H) (n = 24). **(B)** Fold-change gene expression of IBH severity by mean IBH ≤ 100 [mild (M)] (n = 6) and mean IBH > 100 [moderate/severe (M/S)] (n = 28). Fold-change expression comparing mild (M) IBH (IBH-M) to H (column 1), moderate/severe (M/S) IBH (IBH-M/S) (column 2) to H, and IBH-M/S to IBH-M (column 3). The data shown present the fold change as mean ratio per gene of compared groups. The mean was calculated as sum of each individual expression in all horses per gene divided by the number of horses. Fold-change mRNA gene expression color code: green, significant upregulation; light green, non-significant upregulation; orange, significant downregulation; and light orange, non-significant downregulation. mRNA gene expression normalized to GAPDH.

Using qPCR on contemporaneous biopsies showed a significant increase in inflammatory cytokines and chemokines in IBH compared to healthy horses for IL-1α, IL-1β, CXCL8, CCL2, and TNF (**Supplementary Figure S2B**; **Figure 2A**).

In biopsies, an increase in inflammatory CXCL8 and CCL2 (**Figures 1A**, **2B**) was found in both severity groups when compared to healthy controls. For IL-1α, IL-1β, and TNF (**Figures 1A**, **2B**), there was only a significant difference when comparing healthy to moderate/severe IBH.

#### 3.2.2 Eosinophil chemotactic genes increase in skin of IBH horses

Also, an increase in mRNA expression levels of CCL11, CCL26, and CXCL10 (**Supplementary Figure S2B**; **Figure 2A**), all chemoattractants involved in the recruitment of eosinophils to the lesion site, was found. Further upregulation was found for the associated receptors IL-5RA and CCR5, as well as for the eosinophilic master regulator IL-5, when comparing IBH to healthy skin punch biopsies (**Supplementary Figure S2B**; **Figure 2A**).

When taking IBH severity into account, an increase in eosinophil counts associated with CXCL10 was found for both severity groups compared to healthy controls (**Figures 1B**, **2B**), while CCL11 and CCL26 (**Figures 1B**, **2B**) continued to exhibit significantly increased levels in moderate/severe IBH when compared to healthy samples. IL-5Ra and CCR5 not only showed a significant increase in mRNA expression compared to healthy controls but also compared to milder IBH cases (**Figures 1B**, **2B**). Also, a significant increase in CCL24 (**Figures 1B**, **2B**) was found in mild compared to healthy or moderate/severe IBH horses. No significant difference for CCR3 was observed (**Supplementary Figure S3**; **Figure 2B**).

#### 3.2.3 Increase in Th1/Th2/Treg cytokines in skin of IBH horses

Furthermore, mRNA expression of Th2 cytokines such as IL-4, IL-13, and IL-5 (**Supplementary Figure S2C**; **Figure 2A**) significantly increased in IBH skin punch biopsies compared to healthy samples. An increase in IL-31 expression (**Supplementary Figure S2C**; **Figure 2A**) in the lesional skin of IBH compared to healthy horses is associated with Th2-mediated pruritus via the IL-31 signaling axis (20). Interestingly, Th1 cytokines such as IFN-γ and IL-12b significantly increased in IBH skin punch biopsies as well, compared to healthy biopsies (**Supplementary Figure S2C**; **Figure 2A**), as did Treg-associated IL-10 (**Supplementary Figure S2C**; **Figure 2A**).

Regarding gene expression in biopsies and IBH severity, only Th1-associated IFN-γ (**Figures 1C**, **2B**) increased in moderate/severe cases when compared to both healthy and mild IBH horses, whereas Th2-associated IL-13 and IL-5, Th1-associated IL-12b, and Treg-associated IL-10 (**Figures 1C**, **2B**) increased when comparing moderate/severe IBH horses to healthy controls only. Th2-mediated IL-31 expression (**Figures 1C**, **2B**), associated with pruritus, was found to be significantly increased when comparing mild IBH to healthy controls or to moderate/severe IBH. No significant difference was found for IL-4 (**Supplementary Figure S3A**; **Figure 2B**).

#### 3.2.4 Increase in IgE and histamine receptors in IBH horses

Moreover, mRNA levels of IGHE and histamine receptors (HRs) HRH2 and HRH4 (**Supplementary Figure S2C**; **Figure 2A**) significantly increased in IBH skin punch biopsies, compared to healthy controls, whereas neither HRH1 nor HRH3 was expressed in any group (data not shown).

A significant increase in IGHE mRNA expression (**Figures 1C**, **2B**) was found in biopsies of both IBH severities when compared to healthy controls. Regarding HRH2 and HRH4 (**Figures 1C**, **2B**), both histamine receptors showed a significant increase exclusively in moderate/severe IBH when compared to healthy and mild IBH horses.

#### 3.2.5 Innate barrier damage and immune-mediated link to the nervous system in skin of IBH horses

Innate barrier damage was indicated in allergic horses by thymic stromal lymphopoietin (TSLP) and IL-33 mRNA expression (**Supplementary Figure S2C**; **Figure 2A**), both of which were significantly increased in IBH lesions when compared to healthy skin. Also, an increase of histamine receptor-independent IL-31Ra (**Supplementary Figure S2C**; **Figure 2A**) was observed in IBH skin punch biopsies compared to healthy horses. Considering disease severity, the increase in TSLP only persisted when comparing severe IBH cases to healthy controls, whereas IL-31Ra was increased when comparing moderate/severe IBH horses to healthy controls as well as to mild IBH cases (**Figures 1C**, **2B**). Furthermore, IL-33 showed a significant increase in mild IBH versus healthy horses but was decreased in moderate/severe cases compared to milder disease presentations (**Figures 1C**, **2B**).

### 3.3 Immune cells in blood correlation with mRNA gene expression in skin biopsies

**Figure 3:**
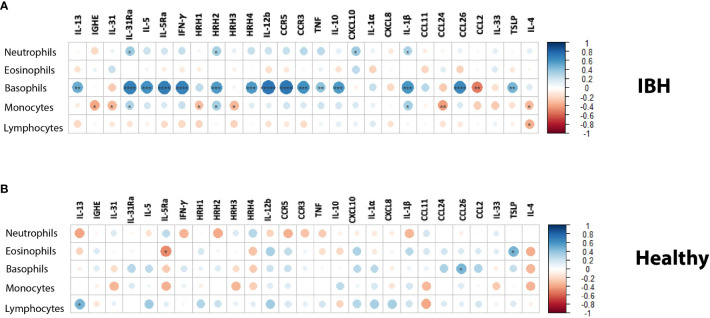
Blood immune cell counts correlation with mRNA tissue gene expression in **(A)** insect bite hypersensitivity (IBH) and **(B)** healthy horses. Statistical significance by Spearman’s correlation is indicated by dot size, from small to larger size increasing with correlation and significance; downregulation in red and upregulation in blue using color gradients. *P < 0.05, **P < 0.01, ***P < 0.001, ****P < 0.0001.

To address the theoretical link between blood cells and tissue markers, the correlation between immune cell differential counts was explored in blood and mRNA gene expression in the tissue of IBH horses. Our data showed a strong and significant positive correlation (r > 0.6) of basophil cell counts in blood with IL-31Ra, IL-5, IL-5Ra, IFN-γ, HRH2, HRH4, IL-12b, CCR3, CCR5, IL-10, IL-1β, and CCL26 in the lesional biopsy skin (**Figure 3A**). In addition, a significant moderate positive correlation (0.4 < r < 0.6) of basophil cell counts was observed in blood with IL-13, TNF, and TSLP in lesional biopsy skin. A moderate and significant negative correlation was found for basophils and CCL2. For the other immune cell counts in blood, our data showed a weak but significant positive correlation of neutrophils with IL-31Ra, HRH2, CXCL10, and IL-1β (**Figure 3A**). In addition, for monocytes, a weak and significant positive correlation was noted with IL-31Ra, HRH2, and IL-1β (r < 4) as well as a weak and significant negative correlation with IGHE, IL-31, HHR1, HRH-3, CCL24, and IL-4 (**Figure 3A**). For lymphocytes, a weak and significant negative correlation was found for IL-4 (**Figure 3A**). Subsequently, the same analysis this time was repeated on the healthy horse blood differential counts and gene mRNA expression in healthy skin biopsies. Among the five immune cells and the 30 genes explored in this study, a weak but significant negative correlation of eosinophils with IL-5Ra and a weak and significant positive correlation of eosinophils with TSLP were observed (**Figure 3B**). For basophils, a weak and significant positive correlation was found with CCL26 (**Figure 3B**). For lymphocytes, a weak and significant positive correlation was found with IL-13 (**Figure 3B**).

### 3.4 Allergen restimulation of PBMCs from healthy and IBH horses

**Figure 4:**
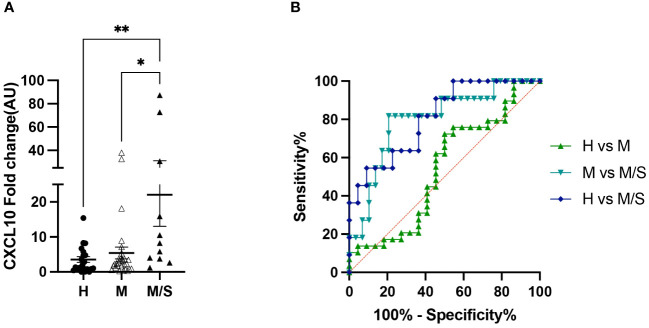
Peripheral blood mononuclear cell (PBMC) restimulation from healthy and insect bite hypersensitivity (IBH) horses with *culicoides* allergen. **(A)** Gene expression of *in vitro* allergen restimulated PBMCs from healthy and IBH horses in relation to disease severity. Gene expression in PBMCs isolated from healthy (H) (n = 22), mild (M) IBH (IBH-M) (n = 29), and moderate/severe (M/S) IBH (IBH-M/S) (n = 11) upon restimulation with *culicoides* whole-body extract. Fold-change expression is shown for *culicoides*-stimulated normalized to unstimulated PBMCs for CXCL10 mRNA expression in mild (M) and moderate/severe (M/S) IBH compared to healthy horses. **(B)** CXCL10 receiver operating characteristic (ROC) analysis from stimulated PBMCs with allergen in healthy and IBH horses. ROC analysis of healthy versus IBH mild and moderate/severe samples for CXCL10 in stimulated PBMCs with culicoides from healthy and IBH horses. *P < 0.05, **P < 0.01.

Concurrent with the differential blood counts, PBMCs were collected for *in vitro* allergen restimulation. Due to the limited availability of sample material, the analyzed gene panel was reduced to the following: IL-1α, IL-1β, IL-4, IL-5, CXCL8, IL-17, IL-31, CCL2, and CXCL10. Of these, only CXCL10 showed a significantly different gene expression, increasing in moderate/severe cases compared to healthy controls as well as to mild IBH cases. Data are presented as fold change normalized to the non-stimulated condition (**Figure 4A**). In addition, the CXCL10 ROC analysis showed high sensitivity/specificity when comparing healthy or mild to moderate/severe allergen-stimulated PBMCs in IBH and healthy horses (**Figure 4B**; **Supplementary Table 3**). Other investigated genes showed no significant changes in the blood (data not shown).

### 3.5 Sensitivity and specificity of severity indicating genes in healthy versus IBH horses

**Figure 5:**
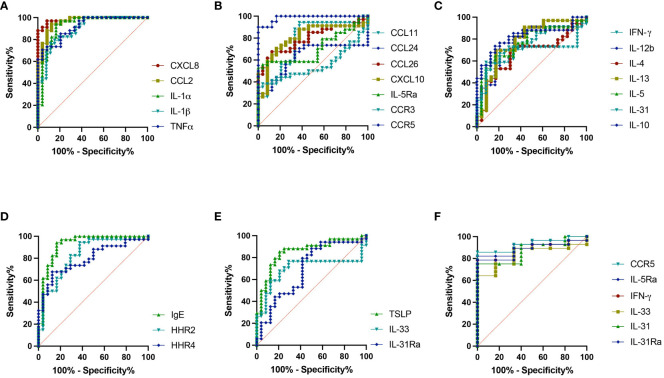
Severity indicating genes. Receiver operating characteristic (ROC) analysis of severity indicating genes from biopsies in healthy and insect bite hypersensitivity (IBH) horses. ROC analyses of **(A)** CXCL8, CCL2, IL-1α, IL-1β, and TNF. **(B)** CCL11, CCL24, CCL26, IL-5Ra, CXCL10, CCR3, and CCR5. **(C)** IFN-γ, IL-4, IL-5, IL-13, IL-12b, IL-31, and IL-10. **(D)** IGHE, HRH2, and HRH4. **(E)** TSLP, IL-33, and IL-31Ra. **(F)** ROC analyses of IL-5Ra, CCR5, IL-31, IL-33, IFN-γ, and IL-31Ra in M vs. M/S horses.

The sensitivity and specificity of all the genes analyzed from skin punch biopsies were also investigated for their potential to accurately predict IBH presentation in horses using ROC analysis. Our investigation showed high and significant sensitivity/specificity (area > 0.8): CXCL8, CCL2, IL-1α, IL-1β, and TNF for the inflammation panel (**Figure 5A**; **Supplementary Table 4**); CXCL10 and CCR5 for the eosinophilic panel (**Figure 5B**; **Supplementary Table 4**); IL-10 for the Th subset panel (**Figure 5C**; **Supplementary Table 4**); IGHE and HRH2 for the IgE panel (**Figure 5D**; **Supplementary Table 4**); and TSLP for the innate barrier panel (**Figure 5E**; **Supplementary Table 4**). Further genes with good and significant sensitivity/specificity (0.7 < area < 0.8) were found: CCL11, CCL26, and IL-5Ra for the eosinophil panel (**Figure 5B**; **Supplementary Table 4**); IFN-γ, IL-12b, IL-13, and IL-5 for the Th subset panel (**Figure 5C**; **Supplementary Table 4**); and HRH4 for the IgE panel (**Figure 5D**; **Supplementary Table 4**). Additional ROC analysis was conducted on the genes differentially expressed in IBH horses comparing mild to moderate/severe disease: IL-5Ra, CCR5, IFN-γ, and IL-31 were significantly upregulated and IL-31 and IL-33 were significantly downregulated in M/S compared to M horses. ROC analysis showed high significant sensitivity/specificity (area > 0.8) (**Figure 5F**; **Supplementary Table 5**) when comparing M/S and M horse groups.

## 4 Discussion

In the current study, we investigated the immune cell counts in the blood simultaneously with the expression of genes in the skin of IBH and age-matching healthy control horses. The focus of genes relates to previously described innate and adaptive immune cells and their biological function in IBH.

Our results reveal an inflammatory monocytosis in the blood that was significantly associated with IBH, in particular with moderate/severe IBH. Along the same lines, earlier, we described a similar pattern of monocytes and eosinophils in blood when observing blood cell counts over a whole IBH season (21). This may suggest somehow a dependence or crosstalk between both immune cells. Our data further show a significant increase in the mRNA expression of inflammatory cytokines and chemokines in lesional IBH skin when compared to healthy skin. In addition, pro-inflammatory cytokines IL-1α, IL-1β, and TNF were associated with severity, while chemotactic factors CXCL8 and CCL2 were rather independent of severity but with high sensitivity and specificity for IBH in general. CCL2 predominantly attracts monocytes to the skin (17, 22), while CXCL8 is involved in basophil, neutrophils, and T-cell recruitment (23–27). Moreover, IgE-binding monocytes have been described in horses and may be linked with allergy by triggering the release of CXCL8 supporting basophil recruitment (28). Overall, the data suggest that inflammation is a general characteristic of IBH, whereas monocytes and associated inflammatory cytokines and chemokines, with a special emphasis on IL-1β and HRH2, may play a role in the enhancement of type I and/or type IV allergic immune responses.

In addition to inflammation, eosinophilia is another well-known hallmark of IBH. Across species, eosinophils are commonly associated with allergy, asthma, and infection and represent an inflammatory and toxic cell type, which causes tissue damage (29). Eosinophil accumulation in IBH horses aligns with our previous findings of a highly significant increase in eosinophil counts in blood yet in a severity-dependent manner (15). Further, we observed an increase in eosinophilic chemokines (such as CCL11, CCL26, and CXCL10) and receptors (such as IL-5Ra and CCR5) in lesional skin punch biopsies from IBH horses compared to healthy controls. In particular, our data show that IL-5Ra and CCR5 were associated with IBH severity, with high sensitivity and specificity for CCR5, CCL26, and CXCL10. Also, CXCL10 was the only gene significantly upregulated in allergen-stimulated PBMCs from moderate/severe IBH compared to healthy and mild IBH horses. CXCL10, also called IFN-γ-inducing protein (IP-10) (30), as such is secreted upon response to IFN-γ by various cells including monocytes and epithelial cells (31–33). Additionally, others demonstrated that CXCL10 upregulation through the CXCR3 axis causes attraction of Th1 cells and activates eosinophils (32, 34, 35). Indeed, our data show an increase in CXCL10 and Th1-associated cytokines IFN-γ and IL-12b in IBH compared to healthy horses. CCR5 is expressed on Th1-type lymphocytes, monocytes/macrophages, basophils, eosinophils, and dendritic cells (DCs) when they are in the immature stage (36) and may play a role in innate immune cell migration, particularly in the migration of eosinophils. CXCL10 and CCR5 have been described earlier in the context of IBH in a study comparing RNAseq data in IL-4/-5/-13 and TNFα-stimulated equine keratinocytes derived from IBH and healthy horses (37). Finally, eotaxins have also been described in the context of IBH and, as human analogs are responsible for the recruitment, accumulation, and activation of eosinophils in the skin (38, 39), with CCL26 potentially being involved in the transepithelial migration of eosinophils (40). Taken together, this suggests that eosinophils in the blood and their recruitment into the skin play a key role in IBH development and progression, exhibiting a strong link with disease severity. Expression of IL-5Ra seemed to negatively correlate with eosinophils of healthy horses. We previously demonstrated that our eIL-5-CMV-TT vaccine successfully induced autoantibodies against eIL-5 and mediated a statistically significant reduction in eosinophil counts in blood and lesion scores in vaccinated horses when compared with placebo horses and the pre-treatment season (15, 41), further solidifying the role of eosinophils as main players in IBH and as promising therapeutic targets. Recently, we identified two subsets of eosinophils in IBH horses with different phenotypes and migration functions: resident (rEos) and inflammatory eosinophils (iEos). rEos are mainly present in low percentages in healthy horses’ blood, while iEos are dominant in IBH horses, exhibiting increased granular content and high migratory properties compared to rEos (42). The eIL-5-CMV-TT vaccine successfully reduced iEo counts in the blood of IBH horses, suggesting an IL-5 dependency for the development of the iEo phenotype.

The number of lymphocytes in blood was comparable in healthy and IBH horses, whereas several Th1, Th2, and Treg cytokines such as IL-4, IL-5, IL-10, IL-12b, IL-13, IL-31, and IFN-γ, were upregulated in lesional biopsies of IBH horses when compared to healthy horses. The latter cytokines were further associated with a more severe disease presentation and showed good sensitivity and specificity for IBH, with the exception of IL-4 and IL-31. However, Th2 cells are not the only source of IL-4 and IL-13, with basophils (43–45) and mast cells (46, 47) also releasing the cytokines in relatively large quantities, thus making them suboptimal indicators of Th2 involvement. In addition, our data show that Th1 cytokine, IFN-γ, seems to be also involved in IBH immunopathology. In other studies, as in human atopic dermatitis (AD), IFN-γ was described as a driver of chronic inflammation, and its overexpression can lead to recurrent inflammation and pruritus, causing lichenoid degeneration of the skin in the chronic phase (48, 49) and, as such, may also have a role in the lichenification process of IBH. In addition, IL-10, highly expressed in the lesional skin of IBH horses in our data, is another T-cell cytokine that is produced by adaptive immune cells such as Th1, Th2, Th17, Treg, CD8+ T cells, and B cells (50–53). Also, IL-10 could be secreted by innate immune cells such as DCs (54), macrophages (55), mast cells (56), natural killer (NK) cells (57), eosinophils (58), and neutrophils (59). Interestingly, IL-10 showed high sensitivity and specificity, implicating potential T-cell exhaustion in IBH-affected skin. The role of IL-10 in IBH, however, needs further investigation, as it may be secreted by several adaptive and innate immune cells, which all play important roles in skin allergy (60, 61). A potential link between IL-10 and eosinophilia may be supported by data from IL-10^−/−^ mice showing diminished skin infiltration of eosinophils and IL-4 and IL-5 mRNA expression in a mouse model of allergic dermatitis (62). Recently, Th2 cells, a major source of IL-4, IL-5, and IL-13, were subdivided into conventional Th2 (cTh2) cells and pathogenic effector Th2 (peTh2) cells (63). cTh2 cells mainly express IL-4 and IL-13, thereby promoting the B-cell class switch to IgE antibody-producing plasma cells in type I allergic reactions. Also, cTh2 cells are suggested to produce only low levels of IL-5 and hence play a less decisive role in recruiting eosinophils as what occurs in the late phase of type I and delayed type IVb hypersensitivity allergic reactions (63). In contrast, peTh2 cells are highly positive for IL-5 and thus a major driver of eosinophil production, recruitment, and activation leading to eosinophil-mediated tissue damage. It has been suggested that chronic antigen exposure promotes the transformation from cTh2 cells into peTh2 cells. Indeed, our data showed high and strongly significant levels of IL-5 and IL-5Ra that were expressed in severe IBH skin punch biopsies compared to healthy or mild IBH horses.

During allergen sensitization, Th2 interaction with B cells leads to the production of culicoides-specific IgE that binds to its receptor FcϵR1 on the membrane of mast cells and basophils (64). A re-encounter with an allergen provokes degranulation and secretion of mediators such as histamines and leukotrienes, which initiate clinical manifestations of allergy. Basophils, as well as eosinophils, also express IL-5 receptor (65) together with receptors for IL-33 (66–68) and TSLP (69, 70). As such, their role is not limited to IgE-mediated reactions through FcϵR1, but they also infiltrate inflammatory lesions and release pro-inflammatory mediators like histamines and leukotrienes, as well as cytokines IL-4 and IL-13. Our results showed a significant increase in basophil cell counts together with an increase in IgE, HRH2, and HRH4 mRNA expression in IBH skin, both of which are associated with IBH disease emergence and severity, with IgE and HRH2 showing the highest sensitivity and specificity. Concerning the link between different allergic immune cells, we previously showed that eIL-5-CMV-TT vaccination significantly decreased the number of inflammatory eosinophils in blood but also reduced basophil cell counts in the second vaccination year, suggesting a long-term and broader dampening of allergy upon vaccination (21, 42). Strikingly, correlating gene expression in skin and blood cell counts revealed strong and positive correlation with basophils and genes involved but not limited to the eosinophilic panel such as IL-5, IL-5Ra, CCR5, CCR3, and CCL26. As such, this may point to a direct bystander effect of IL-5 on basophils, which express IL-5Ra (71, 72). Other top candidates that positively correlated with basophil counts were IL-31Ra, IFN-γ, and IL-1β, while IL-1β also correlates to a weaker extent with neutrophil and monocyte cell counts.

TSLP and IL-33 are two alarmin cytokines that drive type 2 responses at the skin barrier level and hence play a role in the early development of allergic reactions (73, 74). Both are potential targets in the treatment of allergic diseases (75). Indeed, TSLP and IL-33 were both expressed in lesional IBH biopsies and levels correlated with disease severity with high sensitivity and specificity. Also, TSLP has been confirmed in earlier studies to play an important role in IBH by mRNA quantification either in lesional IBH biopsies (17) or in *culicoides* allergen-stimulated equine primary keratinocytes (37, 76, 77). The scratching of insect bites seemed to cause early skin barrier defects and related TSLP upregulation, which may be important in priming a type 2 response. Th2-related peripheral pruritus mediated by IL-31 acting on the specific receptor IL-31Ra on peripheral nerves (17) and other immune cells such as eosinophils (78–80) seemed to be involved and was associated with a more severe disease presentation. The expression of IL-31Ra was significantly elevated in acute lesions, whereas IL-31 was significantly expressed in mild when compared to acute and healthy skin horses.

In summary, our results indicate that IBH-affected horses have multifactorial strong inflammatory responses, impaired T-cell cytokines, and increases in eosinophils, monocytes, and basophils when compared with healthy controls. Interestingly, a set of genes such as CXCL8, CCL2, CXCL10, and IgE was expressed to be similarly upregulated in both mild and moderate/severe when compared to healthy horses. By contrast, IL-5Ra, CCR5, IFN-γ, and IL-31Ra were significantly expressed only in severe IBH compared to mild and healthy horses. In addition, IL-31 and IL-33 were significantly upregulated genes in mild IBH horses compared to healthy and moderate/severe IBH horses. Interestingly, basophil cell count in blood together with IL-5Ra, CCR5, IFN-γ, and IL-31Ra mRNA expression in tissue not only significantly increased in moderate/severe IBH compared to mild and healthy horses but also showed a strong correlation. In summary, those genes may serve as biomarkers to monitor the onset and progression of IBH in the future. Once confirmed, those new biomarkers can prove invaluable for the detection and monitoring of disease onset and progression as well as for the evaluation of the efficacy of new therapeutic treatments in IBH skin allergy.

## Data availability statement

The original contributions presented in the study are included in the article/**Supplementary Material**. Further inquiries can be directed to the corresponding author.

## Ethics statement

The animal studies were approved by Cantonal veterinary authorities:License 33558. The studies were conducted in accordance with the local legislation and institutional requirements. Written informed consent was obtained from the owners for the participation of their animals in this study.

## Author contributions

FJ: Writing – review & editing, Writing – original draft, Visualization, Validation, Software, Methodology, Investigation, Formal analysis, Data curation. AC: Writing – review & editing, Writing – original draft, Methodology. MD: Formal analysis, Writing – review & editing. SP: Writing – review & editing, Writing – original draft. JL: Methodology, Writing – review & editing, Writing – original draft. TR: Resources, Writing – review & editing, Writing – original draft. GK: Methodology, Writing – review & editing, Writing – original draft. NW: Resources, Writing – review & editing, Writing – original draft. FC: Methodology, Writing – review & editing, Writing – original draft. AF-G: Writing – review & editing, Writing – original draft, Visualization, Validation, Supervision, Software, Resources, Project administration, Investigation, Funding acquisition, Formal analysis, Data curation, Conceptualization.
