# Supplementary material for: Cytokines and chemokines skin gene expression in correlation with immune cells in blood and severity in equine insect bite hypersensitivity
Source: Front Immunol. 2024 Jul 15;15:1414891. doi: 10.3389/fimmu.2024.1414891 (PMC11284025; doi:10.3389/fimmu.2024.1414891)
Supplement: Supplementary file 1 [file DataSheet_1.pdf]

Supplementary Material

1 Supplementary Figures and Tables

1.1 Supplementary Figures

1.1.1 Supplementary Figure 1: Lesion score of IBH horses.

Supplementary Figure 1: Lesion score of IBH horses. IBH lesion score measured from April to October in IBH horses.

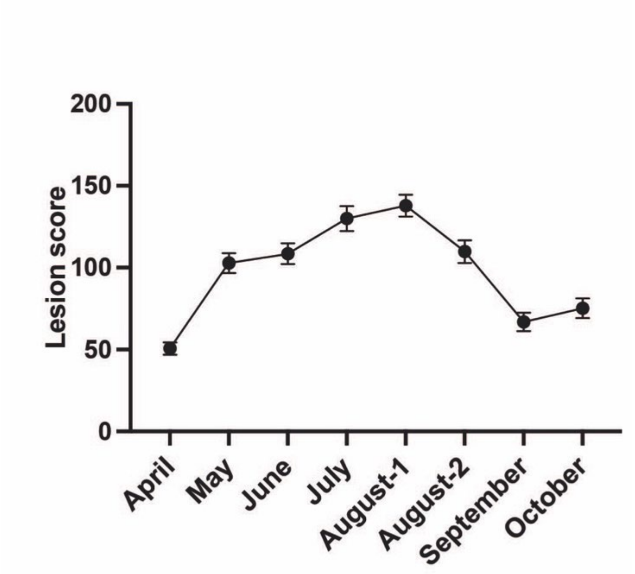

1.1.2 Supplementary Figure 2: Differential blood counts and gene expression in skin punch biopsies from healthy and IBH horses.

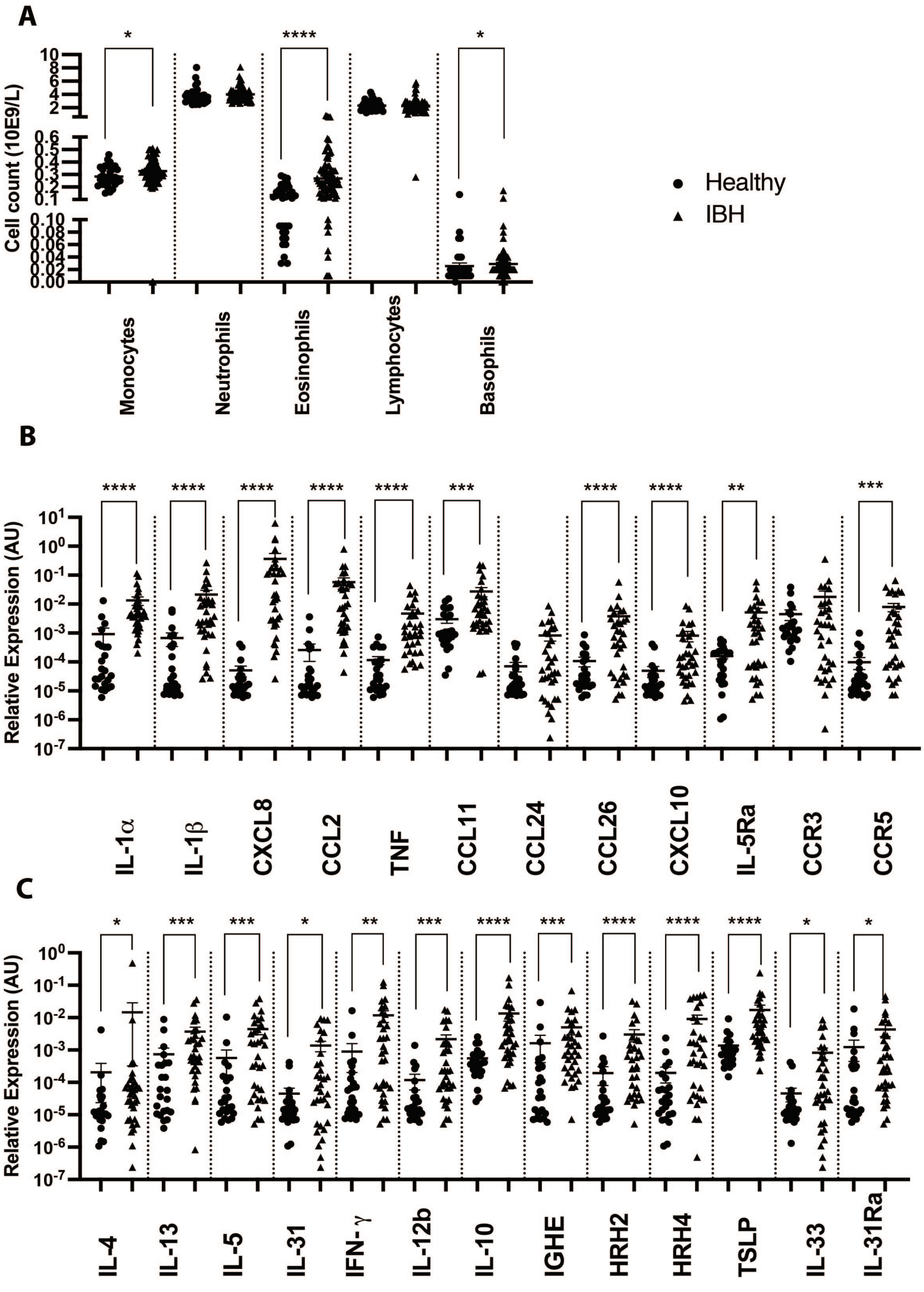

Differential blood cells and skin punch biopsies from healthy skin (H) from healthy horses (n=24 biopsies, n=33 blood) and IBH horses (n=34 biopsies, n=80 blood) with IBH lesion (L) (n=34) biopsies were collected simultaneously during IBH season with high symptoms in August. **(A)** Counts of monocytes, neutrophils, eosinophils, lymphocytes, and basophils in blood of healthy and IBH horses. **(B)** Relative mRNA expression of IL-1α, IL-1β, CXCL8, CCL2, TNF, CCL11, CCL24, CCL26, CXCL10, IL-5Ra, CCR3, and CCR5. **(C)** Relative mRNA expression of Th1, Th2, Treg related genes IL-4, IL-13, IL-5, IL-31, IFN-γ, IL-12b, IL-10, IgE, histamine receptor (HR) H2, HRH4, TSLP, IL-33, and IL-31Ra.

1.1.3 Supplementary Figure 3: Gene expression of IL-4 and CCR3 in skin punch biopsies from healthy and IBH horses in relation to disease severity.

Supplementary Figure 3: Gene expression of IL-4 and CCR3 in skin punch biopsies from healthy and IBH horses in relation to disease severity. Skin punch biopsies were processed for total RNA extraction and qPCR. mRNA expression for gene of interest is normalized to GAPDH (endogenous gene). Relative gene expression presented as $2^{\wedge-\Delta ct}$ (individual quantification, Δct= Ct gene of interest - Ct GAPDH).

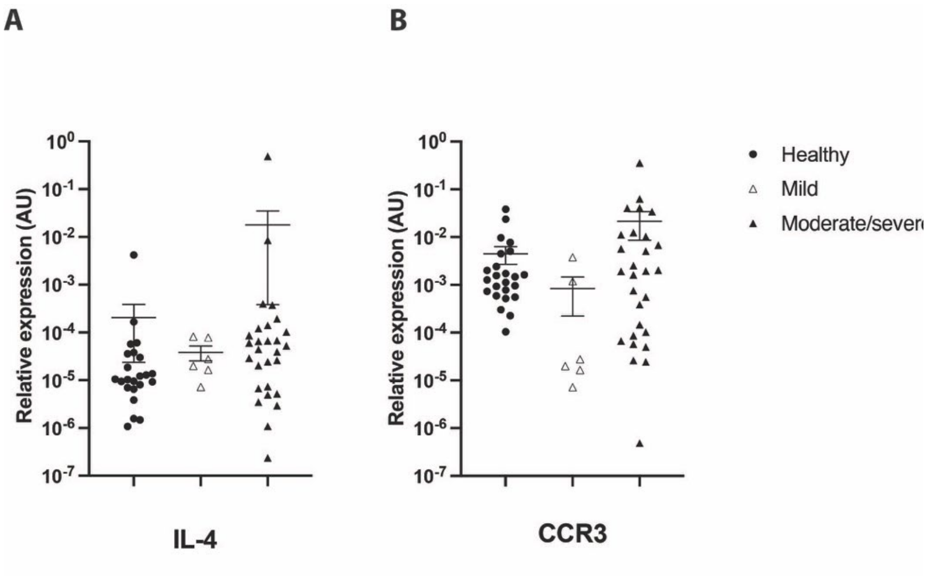

1.1.4 Supplementary Figure 3:

A

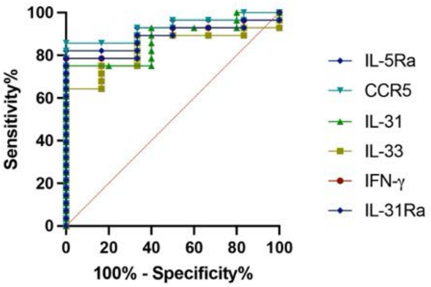

B

Gene/AUC	Area	Std.Error	95% Confidence interval	P Value
**IL-5Ra**	0.881	0.05849	0.7663	0.0038
**CCR5**	0.9286	0.04429	0.8418	0.0011
**IL-31**	0.8714	0.06691	0.7403	0.009
**IL-33**	0.8333	0.07149	0.6932	0.0114
**IFN-γ**	0.881	0.05849	0.7663	0.0038
**IL-31Ra**	0.9107	0.05095	0.8109	0.0018

1.2 Supplementary Tables

1.2.1 Supplementary Table 1: *Location of skin punch biopsies collected from healthy and IBH horses*

Location of the biopsy / Group	Mane/ Mane crest	Head/ between ears	Breast/ elbow front legs	Flank	Knee/Axilla	Total
**Healthy, n=**	24	0	0	0	0	24
**IBH-Lesional, n=**	16	3	6	4	5	34

1.2.2 Supplementary Table 2: List of primer sequences used in this study.

Genes	Sense primer (5'-3')	Antisense primer (5'-3')	Accession Number
GAPDH	ATTGCCCTCAACGACCACTT	TCTTGCTGGGTGATTGGTGG	NM_001163856
HRH1	TTGATCGCTATCGCTCGGTC	CACTTGTCCTCCTGGCGATT	NM_001081919
HRH2	AGCCTCCTCATGTTTGGACG	TCCTAGGACCCTACAGCACC	XM_023617200
HRH3	TTCCTGTCAGTCACTCGTGC	CTGCCACCGGACAGATACTC	XM_023626869
HRH4	AGAGTCTTGGAAGGAGACGGA	GCCACTGAGAAGACCGGAAC	NM_001163969
IL-1α	TGCCTGACACACCCAAAACT	CAACTTTGGATGGGCAACCG	NM_001082500
IL-1β	ATGGGGGCATCCAGCTTCAAT	TGAGCAGGGAACGGGTATCTT	NM_001082526
IL-4	TGGCCCGAAGAACACAGATG	CTTGAGGTTCCTGTCCAGTCC	NM_001082519
IL-5	CTCTTGGAGCTGCCTACGTC	CCCATCGCCTATCAGCAGAG	NM_001082499
IL-5Ra	CAGGCACAGACACGATGGAC	GGCTCTGGCGATCCTCTAGT	XM_023621284
CXCL8	ACCCCCATACAAAGTGGGTG	CATGGATTTTGCCCCTCAGC	NM_001083951
IL-10	CTAGGGAACGAAGCATCCAGG	TCAGGAGAGAGGTACCACAGG	NM_001082490
IL-12b	ATGTGTCACCAGTGGTTGGT	AGGGGTATTGCAGGTGAGGA	NM_001082516
IL-13	TCATCATTTGCGGAGGCAGA	CAGGCTGAGGTCCAAGCTAA	NM_001143791
IL-17a	CTCCACCTCCCCTTGGAATC	ACCTTCCCTTCGGCATTGAC	NM_001143792
IL-31	CCTGTTCCTGCTCTGCTGTT	GACGGCAAGTCACTGTCGAA	XM_023648068
IL-31Ra	CCCCCTCCTGCTTGTGTTAG	GGCCTCGGTGAGAGCTTATC	XM_023625297
IL-33	GGTCCAATTTTTGAAGATTTTGCT	ACTGAGAAGGATAGCAACGGA	XM_001492329
CCR3	GCGGGATGAGTGGGTTTTTG	GGTTCGAAGGGCAAACACAG	XM_005600755
CCR5	GCAAAAGCTGTCCCGTCTTC	TAAAAGAACTGCTCCCAGCCC	NM_001091534
CCL2	AGCATTCCCTAAATGCCCCC	GCAAGGACCCTCAACACCAT	NM_001081931
CCL11	CGTCTGCTGCTTTAACGTGG	TTCTGGGGACATTTGCTGCT	NM_001081871
CCL24	TCAACCTGCAGACACACTCG	TAGCTGTTGGCAGGTCATCC	XM_014729919
CCL26	AGGGACGGGTTAGCCCATAA	GCTCTTCATGGTGCTGCAAG	XM_003362700
CXCL10	TCCAGAGTCCAAGACCGTCA	ACTTCTCTCAGTGTTCGAGGAG	NM_001114940
TNF	CCTACCCGTCCAAGGTCAAC	CATACCAGGGCTTGGCTTCA	NM_001081819
IFN-γ	GATCTGAAGGTCCAGCGCAA	TCCGGCCTCGAAATGGATTC	AF361542
TSLP	CGAGCGCCTTACCTTCAAGT	GCAATGGCTGTTGAGCGTAG	NM_001164063
IGHE	CTCGTCAAGGGCTACTTCCC	AGGTTTGGTCAAAGACGGCA	L31502

1.2.3 Supplementary Table 3: CXCL10 ROC analysis from stimulated PBMCs with allergen in healthy and IBH horses.

CXCL10 fold change normalized to non-stimulated PBMCs in healthy and IBH (M, M/S) plotted for ROC analysis.

Comparaison/AUC	Area	Std.Error	95% Confidence interval	P Value
H vs M	0.5408	0.08515	0.3739	0.621
M vs M/S	0.7962	0.07975	0.6399	0.0042
H vs M/S	0.8099	0.0771	0.6588	0.0042

1.2.4 Supplementary Table 4: Table summarizing ROC analysis of all genes presented in this study.

Table summarizing ROC analysis of all the genes presented in this study.

Gene/AUC	Area	Std.Error	95% Confidence interval	P Value
**CCL2**	0.9608	0.02284	0.916	<0.0001
**CXCL8**	0.9853	0.01141	0.9629	<0.0001
**IL-1α**	0.9265	0.03879	0.8504	<0.0001
**IL-1β**	0.8948	0.04026	0.8159	<0.0001
**TNF**	0.9167	0.03417	0.8497	<0.0001
**CCL11**	0.7696	0.06546	0.6413	0.0005
**CCL24**	0.6287	0.07557	0.4806	0.0974

**CCL26**	0.7953	0.05842	0.6809	0.0001
**CXCL10**	0.8015	0.06065	0.6826	0.0001
**IL-5Ra**	0.7108	0.06842	0.5767	0.0066
**CCR3**	0.5478	0.07688	0.3971	0.5381
**CCR5**	0.9854	0.013	0.9599	0.0001
**IFN-γ**	0.7402	0.06552	0.6118	0.002
**IL-12b**	0.7831	0.06257	0.6604	0.0003
**IL-4**	0.6662	0.07357	0.522	0.0345
**IL-13**	0.7787	0.0659	0.6495	0.0004
**IL-5**	0.7659	0.06348	0.6415	0.0006
**IL-31**	0.6806	0.07441	0.5347	0.0208
**IL-10**	0.8186	0.05614	0.7086	0.0001
**IGHE**	0.9265	0.03879	0.8504	0.0001
**HRH2**	0.8248	0.05707	0.7129	<0.0001
**HRH4**	0.7929	0.05857	0.6781	0.0002
**TSLP**	0.8554	0.05114	0.7552	0.0001
**IL-33**	0.6973	0.07263	0.5549	0.011
**IL-31Ra**	0.69	0.07281	0.5472	0.0144

1.2.5 **Supplementary Table 5: Table summarizing ROC analysis of the genes differentially expressed in mild vs moderate/severe IBH horses.** IL-5Ra, CCR5, IFN-γ and IL-31Ra were significantly upregulated and IL-31, and IL-33 were significantly downregulated in moderate/severe when compared to mild IBH lesion skin punch bopsies horses.

Gene/AUC	Area	Std.Error	95% Confidence interval	P Value
**IL-5Ra**	0.881	0.05849	0.7663	0.0038
**CCR5**	0.9286	0.04429	0.8418	0.0011
**IL-31**	0.8714	0.06691	0.7403	0.009
**IL-33**	0.8333	0.07149	0.6932	0.0114
**IFN-γ**	0.881	0.05849	0.7663	0.0038
**IL-31Ra**	0.9107	0.05095	0.8109	0.0018
